# Childhood lead exposure and sleep problems in adolescents: a longitudinal cohort study

**DOI:** 10.1007/s00420-024-02099-3

**Published:** 2024-09-14

**Authors:** Ying Dai, Olivia M. Halabicky, Xiaopeng Ji, Jianghong Liu

**Affiliations:** 1 School of Nursing, University of Pennsylvania, 418 Curie Blvd., Room 426, Claire M. Fagin Hall, Philadelphia, PA 19104-6096 USA; 2 School of Public Health, University of Michigan, Ann Arbor, USA; 3 School of Nursing, College of Health Sciences, University of Delaware, Newark, USA

**Keywords:** Adolescent, Cohort study, Lead, Sleep problems

## Abstract

**Purpose:**

Childhood lead exposure is linked to poorer neurobehavioral function in adolescence, but the relationship between lead and adolescent sleep health remains inconsistent. This study aimed to investigate concurrent and longitudinal associations between lead exposure and multiple sleep health domains in adolescents.

**Methods:**

A total of 972 adolescents from China Jintan Child Cohort were included in analyses. The Blood lead levels (BLLs) were assessed in two Waves, at ages 3–5 years (mean 6.50 ± 2.76 μg/dL) and 11–13 years (mean 3.12 ± 1.17 μg/dL). Sleep problems at age 11–13 were parent-reported via the Child Sleep Health Questionnaire (CSHQ) and self-reported by adolescents using the Pittsburgh Sleep Quality Index (PSQI).

**Results:**

Both early and later BLLs were associated positively with parental reported sleep problems, including sleep onset delay, night waking, short duration, parasomnias, and disordered breathing. Sex-stratified analyzes showed that most adjusted associations between two-Wave BLLs and sleep outcomes (CSHQ and PSQI) remained statistically significant in males, with a minor increase in the magnitude of these associations. The association between Wave II BLLs and shorter self-reported sleep duration was only statistically significant in female adolescents. Compared to children with consistently low BLLs at both ages, those with persistently high BLLs at both ages had significantly shorter parental-reported sleep duration and worse sleep onset delay.

**Conclusion:**

Findings suggest that both early and later childhood lead exposures link to more adolescent sleep problems, with recent BLLs showing stronger associations with poor adolescent sleep health reported by their parents.

**Supplementary Information:**

The online version contains supplementary material available at 10.1007/s00420-024-02099-3.

## Introduction

Despite progress in eliminating overt sources of lead contamination, lead exposure remains a significant environmental risk factor contributing to the global burden of cardiovascular disease mortality in adults and intelligence quotient (IQ) loss in young children (Larsen & Sánchez-Triana 2023). The United Nations International Children’s Emergency Fund (UNICEF) estimates that 1 in 3 children—up to 800 million around the world—have blood lead level (BLL) $\ge 5 \mu g/dL$, a level that the World Health Organization (WHO) stated that requiring global and regional interventions (Rees et al. 2020). In China, although there has been a significant overall decrease in BLLs in Chinese children over the past 30 years, the geometric mean of BLL remains high (9.51 $\mu g/dL$) and there was a rebound during 2016 and 2017 (Zhang et al. 2020). Adolescents are particularly vulnerable to lead’s neurotoxic effects due to higher absorption compared to adults and critical brain development and maturation across early childhood and adolescence (Arnold & Liu 2020; Ramírez Ortega et al. 2021). Previous research reported the long-term effects of early childhood lead exposure on poorer neurocognitive functioning in adolescence (Halabicky et al. 2023; Liu et al. 2014; Lucchini et al. 2019). Less is known whether and how early and concurrent lead exposure is associated with adolescent sleep health–another essential aspect of brain and neural health.

Poor sleep health has also been a public health concern for adolescents. Approximately 30–50% of adolescents worldwide suffer from short, inconsistently timed, and/or difficulty initiating/maintaining sleep (Dai & Liu 2023; Kocevska et al. 2021; Wheaton et al. 2018). Chinese adolescents have higher prevalence-rates of short sleep duration (44.0–55.0% in 6–9th grade) and late bedtime (40.3% in 6th grade and 91.5% in 9th grade) (Yu et al. 2023), and 76.4% of Chinese rural adolescents had sleep problems (Li et al. 2023). Sleep problems, even without diagnosed sleep disorders, are associated with developmental emotion and behavioral problems (Liu et al. 2022), poor mental and physical health (Armstrong et al. 2014; Wang et al. 2016), overweight/obesity (Ji et al. 2022), and neurocognitive functioning (Jianghong Liu et al. 2012a, b). The interplay between developmental changes in sleep regulation (e.g., delayed melatonin onset phase) and social-behavioral factors (e.g., school schedule and screen time) poses a high risk for unhealthy sleep during adolescence (13–18 years old) (Bartel et al. 2015; Crowley et al. 2018). During this special life stage, adolescent sleep may be particularly vulnerable to chemical exposure.

Emerging studies have shown associations between lead exposure and poorer sleep health in adolescents. A recent systematic review found that current studies reported inconsistent findings regarding the relationship between early childhood lead exposure and sleep health among children and adolescents (Wallace et al. 2023). A cross-sectional study among Mexican children aged 6–8 years illustrated that the blood lead concentrations greater than or equal to 10 µg/dL were associated with parent-report later waking time, shorter duration of sleep, and shorter sleep onset (Kordas et al. 2007). A study of cumulative childhood lead levels and sleep during adolescence indicated that younger adolescents with higher cumulative lead level were associated with shorter sleep duration measured via actigraphy (Jansen et al. 2019). However, another longitudinal study found that elevated blood lead levels (BLLs) in early childhood were only associated with increased risk for short sleep duration reported by parents but not adolescents’ self-reports (Liu et al. 2016). These inconsistent findings suggest that the association between lead exposure and children and adolescent sleep health warrants further investigation.

Another gap is that current studies only included early childhood lead exposure and it remains unclear whether the changes in BLLs across early childhood to adolescence also impact adolescent sleep health. Sleep health is a multi-dimensional concept that encompasses appropriate timing, adequate duration, high efficiency, subjective satisfaction, and sustained alertness during waking hours (Meltzer et al. 2021). Comprehensively investigating the impact of lead exposure on these diverse sleep domains is necessary. Furthermore, adolescent self-reported sleep indicators such as sleep duration and sleep latency were subjective to bias (Arora et al. 2013). Given that Chinese parents generally have more involvement in their adolescent offspring’s sleep such as setting bedtime and getup time (Yang et al. 2020), inviting both parents and adolescents to report sleep health is a feasible way to alleviate this self-recall bias. Finally, emergent evidence denotes sex may moderate the effects of childhood lead exposure on adolescent neurocognitive functioning (Halabicky et al. 2022a, b) as well as the association between cumulative social adversity and adolescent sleep duration (Covington et al. 2023). It remains less clear whether there are sex-specific vulnerabilities in the relationship between lead exposure and adolescent sleep. To fill these gaps, this research aimed to explore the association between early childhood and adolescent blood lead exposure and adolescent multi-dimensional sleep problems. Addressing these gaps can facilitate risk stratification and the design of targeted interventions promoting resilient sleep health for those with high lead exposure risks.

## Materials and methods

### Study design and data source

**Fig. 1:**
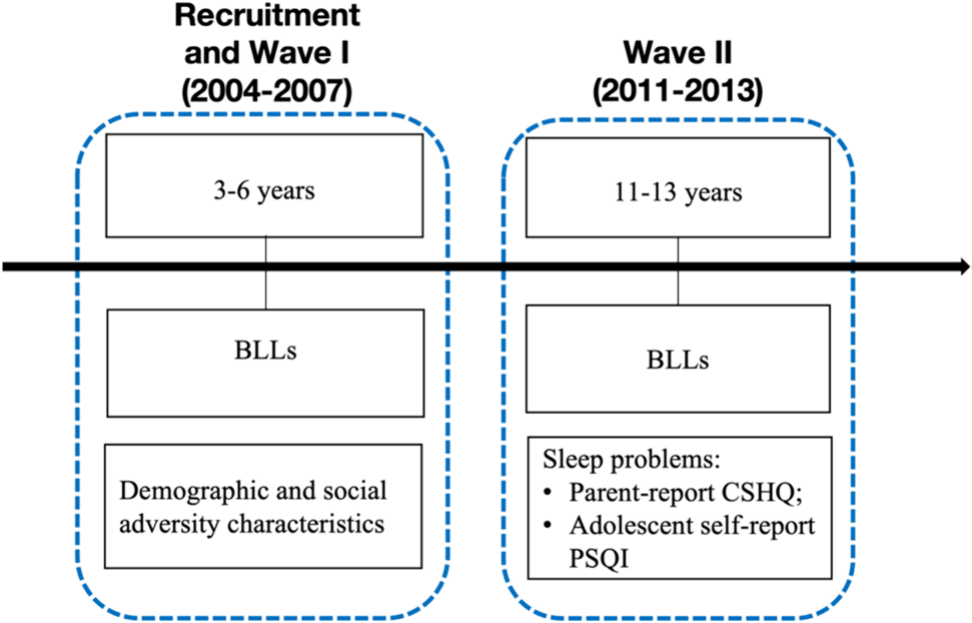
China Jintan Child Cohort (CJCC) data collection process

This longitudinal study was part of the China Jintan Child Cohort (CJCC) study. The CJCC is an ongoing longitudinal preschool cohort designed to examine the effects of lead exposure and micronutrient deficiency on the development of children’s and adolescents’ neurocognitive and neurobehavioral outcomes (Liu et al. 2010). Detailed information about the CJCC is presented elsewhere (Liu, Cao, et al., 2015; Liu et al. 2010). Briefly, during 2004–2005, the CJCC study recruited a total of 1656 preschool children aged from 3 to 5 years old from four pre-schools in Jintan City that were representative of the geographic, social, and economic profile of the city. Later, approximately 1385 and 1100 children participated in Wave I (2005–2007) and Wave II (2011–2013) data collection, respectively. Data collection process are presented in Fig. 1. The current study included a total of 972 children with complete data on confounders and blood lead data in both Wave I and Wave II. Ethical approval has been obtained from the Institutional Review Board of the University of Pennsylvania and the Ethical Committee for Research at Jintan Hospital in China. Written informed consent was obtained from parents at recruitment. During the first wave of data collection (when the children were in preschool), verbal consent was obtained from the children, and written consent was obtained from the parents. During Wave II (when the children were of school age and pre-adolescence), written consent was obtained from both the children and their parents.

### BLLs at 3–5 years and 11–13 years

Baseline BLL was assessed during recruitment (2004–2005) when children were in preschool, and the follow-up BLL was collected at Wave II data collection (2011–2013) when children were 11–13 years old. Fasting venous blood specimens were taken from the children by trained pediatric nurses using lead-free EDTA tubes and following a strict research protocol to avoid lead contamination. Blood specimens were frozen and shipped to the Research Center for Environmental Medicine of Children in Shanghai Jiaotong University for analysis (Liu et al. 2010). The blood specimens were analyzed twice using graphite furnace atomic absorption spectrophotometer, and the final BLL was calculated by the mean of the two measures. Kaulson Laboratories provided blood lead reference materials for quality control (Liu et al. 2015). Detailed information on blood lead collection can be found elsewhere (Halabicky, Pinto-Martin, et al., 2022; J. Liu et al. 2012a, b; Liu et al. 2014).

### Parent-report sleep problems at 11–13 years

Adolescent sleep problems were reported by their parents in Wave II follow-up using the Chinese version of the Child Sleep Health Questionnaire (CSHQ). Parents were asked to recall their child’s sleep behaviors occurring in the past week. The CSHQ consists of 33 items that evaluate children’s sleep health from eight dimensions (i.e., subscales), including bedtime resistance, sleep onset delay, sleep duration, sleep anxiety, night wakening, parasomnias, sleep-disordered breathing, and daytime sleepiness. Each item was rated on a three-point scale: “usually” indicated the sleep behavior occurred 5–7 times per week, “sometimes” for 2–4 times per week, and “rarely” for 0–1 time per week. The sum of all items consists of the CSHQ overall score (ranged between 33 and 99), with higher scores indicating worse sleep problems. The original CSHQ has good internal consistency and excellent discrimination validity (Owens et al. 2000). The Chinese version of CSHQ also demonstrates good internal consistency in the CJCC (Liu et al. 2016).

### Adolescent self-report sleep problems at 11–13 years

Adolescents also reported their sleep problems using the Chinese version of Pittsburg Sleep Quality Index (PSQI) in Wave II follow-up. The PSQI contains 19 items that evaluate adolescent sleep health from seven domains (i.e., subscales): sleep duration, sleep disturbances, sleep latency, sleep efficiency, subjective sleep quality, daytime dysfunction due to sleepiness, and need medication to sleep (Buysse et al. 1989). Each domain ranged from 0 to 3, with higher scores indicating worse sleep health in that domain. Sleep duration was calculated by bedtime and wake-time and classified into 0–3 levels according to the adolescent cut-off used in the National Sleep Foundation (John et al. 2016).The PSQI global score ranged from 0 to 21 (sub-domains range = 0–3), with poor sleeper defined as total scores > 5 (Ramírez Ortega et al. 2021). The Chinese version of the PSQI has been tested in Chinese adolescents and showed excellent internal consistency (Zhou et al. 2012). The internal consistency in the current study was 0.78.

### Confounders

We included adolescent sex, age at baseline blood lead test, residence (city/town/countryside), and household social adversity index as confounders. Recognizing that including too many SES factors in linear modeling can potentially cause multicollinearity, we followed the cumulative risk approach (Evans et al. 2013) and generated a household social adversity index based on ten demographic variables that are representative of the children’s household socioeconomic status and adversity. These variables include: paternal low education (0 = ‘middle school or above middle school’, 1 = ‘below middle school’); maternal low education (0 = ‘middle school or above middle school’, 1 = ‘below middle school’); paternal low occupational status (0 = ‘professional or skilled work’, 1 = ‘unskilled work’, 2 = ‘unemployment’); maternal low occupational status (0 = ‘professional or skilled work’, 1 = ‘unskilled work’, 2 = ‘unemployment’); paternal poor health status (0 = ‘no problem’, 1 = ‘has one or more of the following conditions: mental health problem, alcoholism, poor physical health); maternal poor health status (0 = ‘no problem’, 1 = ‘has one or more of the following conditions: mental health problem, alcoholism, poor physical health); not raised by biological parents (0 = ‘no’, 1 = ‘yes’); house size below 70 square meters (0 = ‘no’, 1 = ‘yes’); and living in disadvantaged neighborhood. Specifically, disadvantaged neighborhood was identified based on the following conditions: overcrowding, noise, dampness, garbage accumulation, frequent quarrels or theft or fight, presence of pests, construction problems, graffiti, and gambling activities. Parents who answered ‘yes’ to any of these questions were categorized as living in a disadvantaged neighborhood, whole those who answered ‘no’ to all questions were categorized as living in a non-disadvantaged neighborhood. The overall social adversity index was generated by summing the scores of each item, with higher scores reflecting higher social adversity. All demographic and neighborhood variables used to compute the social adversity index were collected at first follow-up (2005–2007) when the child was aged 5–6 years.

### Statistical analysis

Descriptive statistics including mean, standard deviation, and percentages were used to describe the characteristics of included adolescents. Spearman correlation was conducted to estimate the bivariate correlation between BLLs and the total and sub-dimension scores of CSHQ and PSQI, respectively. Student t tests and ANOVA were conducted to explore mean differences of CSHQ and PSQI across adolescent sex and BLL categorical groups (see detailed information below). When significant mean differences were found in ANOVA, the post hoc Tukey’s HSD test was further conducted for multiple comparisons.

Both waves of BLLs were log-transformed when examining the association between BLL and sleep problems given the distribution of BLLs were right-skewed. We further dichotomized BLLs in each Wave using medians to describe the changes of BLLs across different waves. Sample median was chosen as the cutoff value because of the following reasons. First, only a small proportion (8%) of CJCC children had BLLs higher than the reference value of 10 $\mu g/dL$ during Wave I and none of these children had BLLs higher than the reference value during Wave II. Second, there was a sharp decrease in Chinese children’s BLLs during the past 30 years and that the latest BLL reference value of 3.5 $\mu g/dL$ was still under review (Li et al. 2020). We assumed the blood lead concentration higher than the cutoff as the children with high BLLs than sample median in our sample. By dichotomizing Wave I BLL by its median (6.20 $\mu g/dL$), and Wave II BLL by its median (2.90 $\mu g/dL$), we classified the trajectory of lead exposure according to the within sample distribution: Low-Low (‘low’ BLLs than sample median at both Waves), Low–High (‘low’ BLL than sample median at Wave I and ‘high’ BLL than sample median at Wave II), High-Low (‘high’ BLL than sample median at Wave I and ‘low’ BLL than sample median at Wave II), and High-High (‘high’ BLL than sample median at both Waves).

**Fig. 2:**
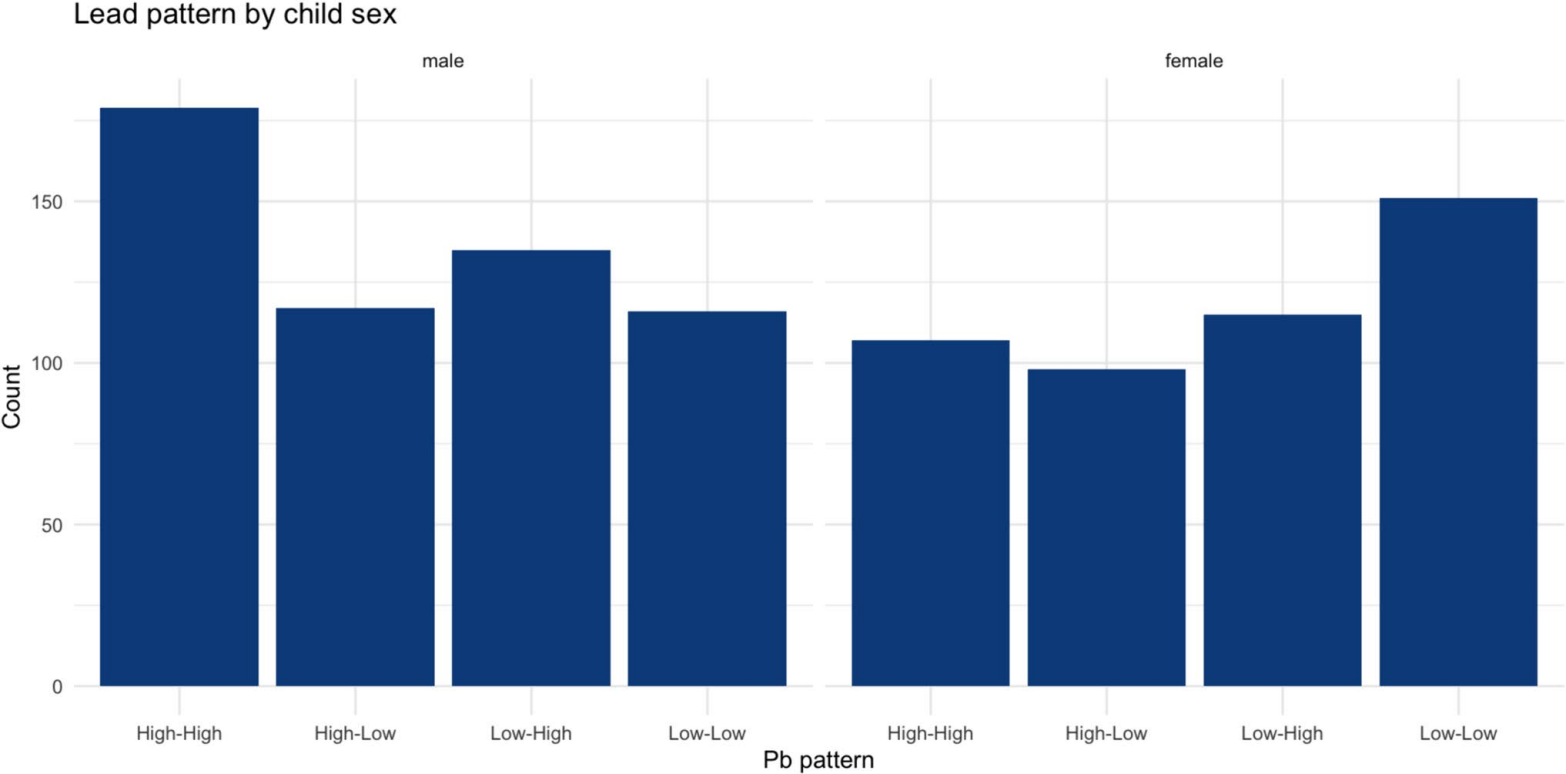
BLL pattern by adolescent sex. High-High: ‘high’ BLLs at both Waves; High-Low: ‘high’ BLL at Wave I and ‘low’ BLL at Wave II; Low–High: ‘low’ BLL at Wave I and ‘high’ BLL at Wave II; Low-Low: ‘low’ BLLs at both Waves

General linear models (GLM) were used to evaluate the adjusted association between BLLs and the overall score and sub-dimension scores of CSHQ and PSQI, respectively. Each Wave of BLLs was separately entered into the linear models to avoid multicollinearity. In preliminary analysis, we found that there was a significant difference in the BLL change pattern across different waves of follow-up between males and females (see Fig. 2), and the association between BLL and CSHQ and PSQI were generally different between males and females (see Supplementary Fig. 1). However, no significant interaction was found between child sex and BLLs in any GLMs. To control for potential sex-based differences, we further stratified the GLMs for males and females after conducting GLMs using the whole sample. Complete case analyses were conducted for all tests since our preliminary analysis showed that missingness in CSHQ was associated with child sex, residency, and social adversity, while missingness in PSQI was associated with Wave I BLLs, child sex, and residency (Supplementary Fig. 1 and Supplementary Table 1). These findings indicate that missingness in CSHQ and PSQI was at random (MAR). Sensitivity analysis was further conducted to compare the results of analysis using non-parametric imputation with the results of analysis using complete case analysis. Nonparametric missing value imputation was conducted using the “missForest” package as the random-forest algorithm does not have assumptions on the distribution of missingness and has excellent predictive power (Stekhoven 2013). All tests were two-tailed and a P-value of less than 0.05 was considered statistically significant. All statistical analyses were conducted using R (version 4.4.0).

## Results

### Demographic characteristics

A total of 972 CJCC adolescents (53.6% males) were included in analysis (Table 1). Males had significantly higher BLLs than females both at Wave I (6.84 ± 3.0 vs 6.11 ± 2.4, t = 5, p < 0.001) and Wave II (3.21 ± 1.17 vs 3.03 ± 1.16, t = 2, p = 0.01). Compared to females, males had higher proportions of high BLLs across preschool and early adolescence (Fig. 2). The mean scores for parent-report CSHQ overall score (44.3 ± 10.2) and adolescent self-report PSQI overall score (5.09 ± 2.55) were both higher than the cutoff scores for sleep problems (CSHQ: 41 and PSQI: 5), indicating that self-report sleep problems were common in CJCC adolescents. Detailed scores for each subscale were presented in Table 1.

**Table 1:** Demographic characteristics of included adolescents

Characteristics	n	mean (SD)
Age at first blood lead test (in months)	972	58.1 (10.8)
Sex, n (%)		
Male	521 (53.6)	
Female	451 (46.4)	
Residence		
City	710 (73.0)	
Town	158 (16.3)	
Rural	104 (10.7)	
Blood lead 3–5 years (mcg/dl)	972	6.50 (2.76)
Blood lead 11–13 years (mcg/dl)	972	3.12 (1.17)
Social adversity at 3–5 years	972	2.26 (1.46)
CSHQ score (parent-report)		
Bedtime resistance	540	8.09 (2.61)
Sleep onset delay	630	1.44 (0.71)
Sleep duration	558	4.31 (1.53)
Sleep anxiety	502	5.55 (1.88)
Night waking	483	3.72 (1.29)
Parasomnia	466	8.74 (2.57)
Sleep disordered breathing	492	3.57 (1.32)
Daytime sleepiness	496	12.1 (3.24)
CSHQ overall score	400	44.3 (10.2)
PSQI score (pre-adolescent self-report)		
Duration of sleep	819	1.18 (0.86)
Sleep disturbance	843	0.90 (0.49)
Sleep latency	864	0.91 (0.85)
Day dysfunction due to sleepiness	841	0.92 (0.83)
Sleep efficiency	775	0.16 (0.50)
Sleep quality	846	0.95 (0.76)
Need meds to sleep	865	0.07 (0.36)
PSQI overall score	710	5.09 (2.55)

### Bivariate correlation between BLLs and sleep problems

**Fig. 3:**
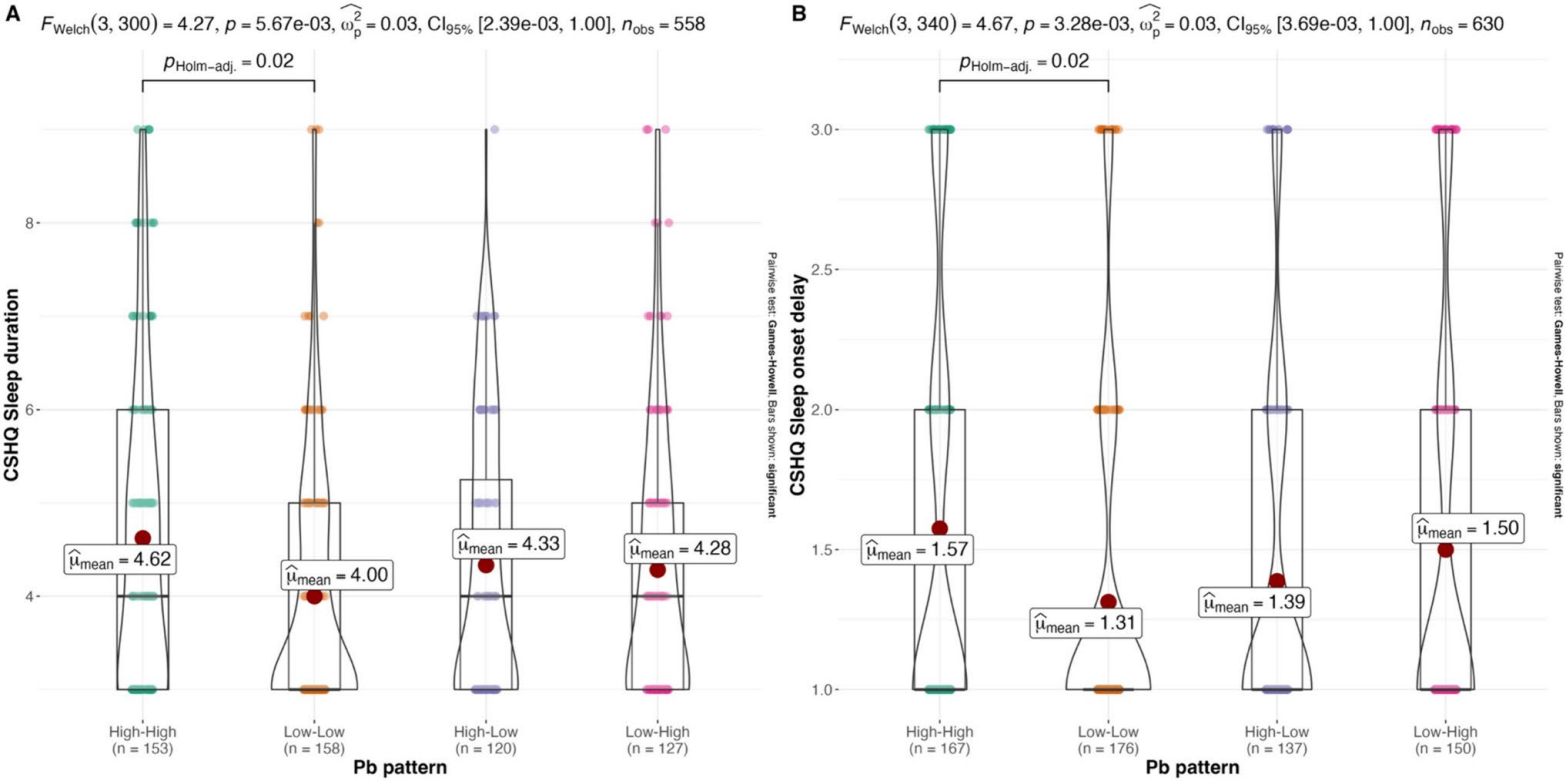
Model-based mean CSHQ (parent-report) sub-dimension scores comparison between groups defined by BLLs patterns. **A** CSHQ (parent-report) short sleep duration scores by BLLs pattern; **B** CSHQ (parent-report) sleep onset delay by BLLs pattern

Spearman correlation showed that Wave I BLLs were positively associated with two CSHQ subscales (sleep onset delay, r = 0.1, p < 0.05, and short sleep duration, r = 0.12, p < 0.01). Increasing Wave I BLLs were also associated with decreasing sleep duration (r = 0.1, p < 0.05) measured by the PSQI. Wave II BLLs were positively associated with one CSHQ subscale: parasomnias (r = 0.11, p < 0.01); and negatively associated with one PSQI subscale (longer sleep latency, r = − 0.06, p < 0.05). Detailed bivariate correlations are presented in Supplementary Fig. 2. Regarding BLL patterns across two Waves, ANOVA tests showed that compared to adolescents who had ‘Low-Low’ BLLs at both Waves, those with consistently ‘high’ BLLs had significantly higher CSHQ short sleep duration and longer sleep onset delay subscales (Fig. 3).

### Multivariable analysis of BLLs and parental reported sleep problems (CSHQ) and youth self-reported sleep problems (PSQI)

Controlling for adolescent age at baseline blood lead tests, social adversity, and residence, total sample analyses showed that BLLs at 3–5 years were positively associated with two CSHQ subscales at 11–13 years: sleep onset delay (β = 0.08, p < 0.01), and night waking (β = 0.15, p < 0.05). Cross-sectionally, BLLs at 11–13 years were positively associated with CSHQ overall score (β = 1.97, p < 0.05) and four sub-scales: short sleep duration (β = 0.17, p < 0.01), night wakings (β = 0.15, p < 0.05), parasomnias (β = 0.36, p < 0.01), and sleep disordered breathing (β = 0.12, p < 0.05).

In GLMs stratified by adolescent sex, most adjusted associations between two-Wave BLLs and CSHQ and PSQI only remained statistically significant in males, with minor increasing in the magnitude of these associations (see Table 2, Female and Male models). In females, higher BLLs at 11–13 years were positively associated with one PSQI sub-scale: shorter sleep duration (β = 0.08, p < 0.05).

**Table 2:** BLL and preadolescent sleep problems at wave II (11–13 years)

	BLL at 3–5 years						BLL at 11–13 years					
	Total		Female		Male		Total		Female		Male	
	β	95% CI	β	95% CI	β	95% CI	β	95% CI	β	95% CI	β	95% CI
Parent-report CSHQ												
CSHQ Overall (n = 400)	0.83	[− 0.17, 1.83]	0.56	[− 0.89, 2.00]	1.22	[− 0.14, 2.58]	1.07*	[0.11, 2.03]	0.59	[− 0.84, 2.02]	1.48*	[0.19, 2.77]
Bedtime Resistance (n = 540)	0.05	[− 0.17, 0.28]	0.08	[− 0.26, 0.42]	0.00	[− 0.31, 0.31]	0.07	[− 0.15, 0.29]	0.12	[− 0.21, 0.46]	0.03	[− 0.27, 0.32]
Sleep Onset Delay (n = 630)	0.08**	[0.02, 0.14]	0.06	[-0.03, 0.14]	0.10 *	[0.02, 0.18]	0.03	[− 0.02, 0.09]	0.04	[− 0.05, 0.12]	0.02	[− 0.06, 0.10]
Short Sleep Duration (n = 558)	0.13	[− 0.01, 0.26]	0.09	[-0.10, 0.28]	0.18	[− 0.01, 0.36]	0.17**	[0.04, 0.30]	0.16	[− 0.03, 0.35]	0.18 *	[0.01, 0.35]
Sleep Anxiety (n = 502)	0.00	[− 0.18, 0.17]	− 0.01	[-0.25, 0.23]	0.00	[− 0.25, 0.25]	0.10	[− 0.07, 0.26]	0.13	[− 0.11, 0.37]	0.03	[− 0.20, 0.27]
Night Waking (n = 483)	0.15*	[0.03, 0.27]	0.13	[-0.05, 0.31]	0.18 *	[0.02, 0.33]	0.15*	[0.04, 0.26]	0.10	[− 0.07, 0.27]	0.19*	[0.04, 0.34]
Parasomnias (n = 466)	0.10	[− 0.14, 0.35]	0.09	[− 0.26, 0.44]	0.15	[− 0.18, 0.48]	0.36**	[0.13, 0.59]	0.23	[-0.11, 0.57]	0.46**	[0.15, 0.76]
Sleep Disordered Breathing (n = 492)	0.02	[− 0.10, 0.14]	0.01	[− 0.16, 0.19]	0.04	[− 0.13, 0.21]	0.12*	[0.01, 0.24]	0.06	[− 0.11, 0.22]	0.19*	[0.03, 0.35]
Daytime Sleepiness (n = 496)	0.05	[− 0.24, 0.35]	0.07	[− 0.33, 0.48]	0.05	[− 0.39, 0.48]	0.12	[− 0.16, 0.40]	− 0.03	[− 0.42, 0.36]	0.23	[− 0.18, 0.64]
Pre-adolescent self-report PSQI												
PSQI overall (n = 710)	0.03	[− 0.16, 0.22]	0.20	[-0.09, 0.48]	− 0.14	[− 0.40, 0.12]	− 0.08	[− 0.27, 0.10]	0.01	[− 0.27, 0.29]	− 0.16	[− 0.41, 0.08]
Short Sleep Duration (n = 819)	0.00	[− 0.05, 0.06]	− 0.01	[− 0.09, 0.07]	0.02	[− 0.06, 0.10]	0.02	[− 0.03, 0.08]	0.08*	[0.01, 0.16]	− 0.02	[− 0.10, 0.05]
Sleep Disturbance (n = 843)	− 0.02	[− 0.06, 0.01]	0.03	[− 0.02, 0.08]	− 0.06 *	[− 0.11, -0.01]	0.00	[− 0.04, 0.03]	0.00	[− 0.05, 0.04]	0.00	[− 0.05, 0.05]
Longer Sleep Latency (n = 864)	− 0.04	[− 0.10, 0.01]	− 0.02	[− 0.11, 0.07]	− 0.07	[− 0.15, 0.01]	− 0.06	[− 0.11, 0.00]	− 0.02	[− 0.11, 0.06]	− 0.09*	[− 0.17, -0.01]
Daytime Dysfunction Due to Sleepiness (n = 841)	0.02	[− 0.04, 0.08]	0.04	[− 0.04, 0.13]	− 0.01	[− 0.09, 0.07]	0.01	[− 0.05, 0.06]	0.00	[− 0.08, 0.08]	0.01	[− 0.06, 0.09]
Lower Sleep Efficiency (n = 775)	0.01	[− 0.02, 0.05]	0.03	[− 0.02, 0.08]	− 0.01	[− 0.06, 0.05]	0.00	[− 0.03, 0.04]	0.00	[− 0.05, 0.05]	0.00	[− 0.05, 0.06]
Lower Sleep Quality (n = 846)	0.00	[− 0.06, 0.05]	0.01	[− 0.06, 0.09]	− 0.02	[− 0.09, 0.06]	0.00	[− 0.05, 0.05]	0.03	[− 0.04, 0.11]	− 0.03	[− 0.10, 0.04]
Use of Sleep Medication (n = 865)	0.00	[− 0.03, 0.02]	0.00	[− 0.03, 0.04]	− 0.01	[− 0.05, 0.03]	0.00	[− 0.03, 0.02]	0.00	[− 0.03, 0.03]	0.00	[− 0.04, 0.03]

All BLLs were log-transformed. All models were based on complete case analysis and controlled for adolescent age at the first blood lead test, adolescent sex, residence, and social adversity index

***p < 0.001, **p < 0.01, *p < 0.05

### Sensitivity analysis for the comparison of completed case analysis and imputed data

Sensitivity analysis showed that the main effects of two-Wave BLLs on CSHQ and PSQI stayed largely the same using either complete case analysis (Table 2) or imputed data (Supplementary Table 1). The effects of wave I BLLs were most consistent on CSHQ sleep onset delay sub-scale but less consistent on short sleep duration or night waking. Specifically, the effects of Wave I BLLs on short sleep duration were statistically significant only in the imputed data analysis but not in complete case analysis. Conversely, the effects of Wave I BLLs on night wakings sub-scale were statistically significantly only in complete case analysis but not in imputed data analysis. Furthermore, the effects of Wave II BLLs on CSHQ overall score, short sleep duration sub-scale score, and parasomnias sub-scale score diminished in imputed data analysis but remained statistically significant.

## Discussion

Few longitudinal studies have investigated the associations between low lead exposure and sleep health in adolescents. We report that early (3–5 years of age) and concurrent (11–13 years of age) exposure to lead were both related to sleep problems among early adolescents. Cross-sectional lead exposure in adolescence was significantly associated with parent-reported sleep anxiety, wake at night, parasomnias, and sleep disordered breathing and female adolescent self-reported short sleep duration. We additionally found that children with BLLs below the sample median in early childhood, but above the sample median in early adolescence (indicative of a “low–high” pattern), and those with high BLLs than the sample median in both Waves (a “high-high” pattern), were at increased risk for sleep problems in early adolescence, compared with children who had low BLLs in both Waves (a “low-low” pattern). This association did not occur among children with high BLLs in Wave I and low BLLs in Wave II. In sex-stratified analyses, males appeared to have more significant effects of BLLs on sleep related problems compared to females, though no significant interactions occurred between sex and BLLs in further analyses. These findings highlight lead exposure, both early childhood and in adolescence, was an important risk factor for adolescent’s sleep disturbance.

Our findings are consistent with those previously found relating lead exposure to sleep disturbances, while addressing some critical gaps in the child and adolescent literature. A recent review of environmental toxicants and sleep quality reported associations between heavy metal exposures and self-reported sleep disturbances, though mostly reported in adult populations (Liu et al. 2021). In adult female soldering workers, BLLs and air lead levels were significantly associated with overall sleep quality measured via the PSQI (Mohammadyan et al. 2019). In a study of male and female workers, BLLs were significantly associated with total scores on the PSQI, Insomnia Severity Index (ISI), and Epworth Sleepiness Scale (ESS) (Sadeghniiat-Haghighi et al. 2011). Sleep problems related to BLLs included difficulty falling and staying asleep as well as waking up too early.

Considering children and adolescents, a previous study reported blood lead concentrations greater or equal to 10 $\mu g/dL$ were associated with later waking time and shorter duration of sleep in 6–8 year olds using a parent reported measure (Kordas et al. 2007). Our study expands on this previous study by including two waves of BLL data as well as examining sleep disturbance into adolescence. We found that having increased BLLs in early life (3–5 years) was associated with shorter sleep duration and BLLs in adolescence were cross-sectionally associated with the increased wake at night. In the CJCC, a previous study indicated that elevated BLLs in early childhood was associated with an increased risk for sleep problems and excessive daytime sleepiness in later childhood (Liu et al. 2015). We expand on previous study in this cohort by including two BLL groups with four levels of high/low BLLs in early childhood and adolescent Waves. We found significant associations between children with low BLL in Wave I and high BLL in Wave II and poor sleep, but this association does not exist among children with high BLL in Wave I and low BLL in Wave II. Given the wide range and unequal distributions of potential lead sources in China, such as soil lead, dust lead, notable emissions from coal combustion, industrial lead pollution, lead-containing tinfoil and tin pots, and folk medicines (Dong & Li 2023; Ying et al. 2018), we speculate that these sources collectively contribute to the lead exposure in Jintan adolescents. In adolescence, one study has examined links between lead exposure (at 1–4 years of age) and objective sleep measures (actigraphy in adolescence), reporting that adolescents with previous high lead exposure slept on average 23 min less than those with lower lead exposure (Jansen et al. 2019). The relationship between lead exposure and sleep disturbances remains understudied compared to other cognitive and health related outcomes. In adolescence especially, when sleep is a critical part of neuro and physical development (Tarokh et al. 2016), understanding these relationships further is necessary.

In our sex-stratified analyses, we found that significant results held for males compared to females. This replicates findings in other lead exposure and health related studies, where males appear to experience more significant effects of lead exposure (Singh et al. 2018). For example, in a study of early childhood lead exposure and growth related outcomes, BLLs were negatively associated with weight-for-age Z score and height-for-age Z score in males, but not females (Zhou et al. 2020). In adolescents, males with higher BLLs were found to score higher on scales of aggression in both proactive and reactive categories compared to those with low BLLs, while females showed no significant associations (Glenn et al. 2023). Examinations of lead exposure and attention deficit hyperactivity disorder (ADHD) have shown increased odds of risk for ADHD with increased lead exposure in males, with a largely attenuated effect in females (Ji et al. 2018). Our results are an initial analysis into the sex-specific differences in relationships between lead exposure and sleep disturbance. Further study is necessary to clarify these relationships and examine potential mechanisms that may contribute to sex differences.

Disruption of sleep, particularly in adolescence, is a predictor of later life physical and mental health. Still, attention needs to be given to the tool used to assess sleep disturbances in adolescents, as studies often rely on parent and/or adolescent self-reports. Some investigations find that parents tend to report idealized versions of their adolescent’s sleep, which suggest significantly longer sleep patterns than the adolescent self-reports or of reports from actigraphy collected data (Short et al. 2013). Others report that children overestimate sleep latency compared to their parents, though both adolescent and parent reported measures appear to be valid (Combs et al. 2019). Our results may have been impacted by the accuracy of parent reported measures, as these appeared to be the most significantly associated with BLLs, though some adolescent reported subscales were significant. More objective measures of sleep, such as actigraphy, in addition to parent and self-reported measures, would be helpful in future work to compare associations. As sleep in the adolescent period relates to a host of physical and mental health outcomes, further research is needed to elucidate associations between lead exposure and sleep problems.

We report significant effects of both early childhood and adolescent BLLs on adolescent sleep disturbances. Still, as blood lead is an acute measure of exposure with the half-life of lead in the blood ~ 1 month, we are unable to report on the true cumulative effects of lead exposure on sleep problems. Novel biomarkers of cumulative lead exposure are being developed, such as DNA methylation biomarkers, which reflect life time exposure to lead and could be instrumental in assessing associations with health and behavior related outcomes (Colicino et al. 2021). Cumulative methods in children include measuring dentin lead levels which can estimate prior lead exposure over the prenatal and early childhood period (Arora et al. 2014). In children, cumulative lead exposure from 1 to 4 years of age has been linked to young adult (Mean age 21.4 years) elevated liver enzymes and hepatic triglycerides (Betanzos-Robledo et al. 2021). As methods continue to develop, future research should consider cumulative lead exposure as it relates to sleep disturbance throughout childhood and adolescence into young adulthood as well as testing the mediating role of sleep between lead exposure and neurocognitive and behavioral outcomes.

### Strengths and limitations

As a longitudinal study, the strengths of the study included the use of a prospective design, following children from early childhood to preadolescence. Sleep data was collected from a parent-reported as well as adolescent reported sleep questionnaires to reduce recall bias. Lead data were measured in early childhood and in preadolescents separately, which obtained the longitudinal lead data and allowed for the assessment in BLL change. Despite these strengths, this study had some limitations. The sleep variables were measured by a parent- and adolescent- reported questionnaires which are subjective. Objective measures, such as actigraphy, should be employed to validate subjective measures in future studies. The questionnaires also did not include behavioral and physiological measures of sleep which are more reliable and not subject to recall bias. Although positive relationships were found between BLLs and sleep disturbance, we cannot assume causality and other factors may contribute to this association or influence sleep behaviors. Moreover, cultural influences and napping could be uncontrolled confounders for our study, as children’s sleep habits can be shaped by cultural context. While we did include two time points of lead exposure, BLLs are an acute measure of lead exposure and more cumulative measures are needed to assess the impact of lead exposure over time on sleep disturbance outcomes. As a result of these limitations, we acknowledged the need to interpret our conclusions carefully.

## Conclusion

In this longitudinal cohort study, findings indicated that both early childhood and adolescent lead exposures were associated with increased risk for sleep problems in both parent and adolescent reported sleep scales, where high adolescent BLLs seemed to be more predictive of sleep disturbance. Further, adolescents who had high BLLs in early childhood and adolescence, compared to those with low BLLs across time points, were found to have more sleep disturbances in adolescence. Males also appeared to have greater BLLs and sleep disturbance associations compared to females. This is an important advancement in the understanding of BLLs impact children’s sleep problems from the perspective of time and in identifying contributors to improve sleep-related health outcomes. Lead pollution is pervasive throughout many developing and developed countries. While the lead exposure rates are declining due to the rise of public awareness, it still presents a significant health impact for children. Results of this study should be used to support policy change for a generation of new strict lead emissions standards, highlighting the hazards of lead exposure to the public, and promoting the public health departments to take further actions to reduce lead exposure.

## Supplementary Information

Below is the link to the electronic supplementary material.

Supplementary file1 (DOCX 836 KB)

Supplementary file2 (CSV 3 KB)

## Data Availability

The data that support the findings of this study are available from the corresponding author, JL, upon reasonable request.
