# Supplementary material for: Childhood lead exposure and sleep problems in adolescents: a longitudinal cohort study
Source: Int Arch Occup Environ Health. 2024 Sep 14;97(9):959–70. doi: 10.1007/s00420-024-02099-3 (PMC11561101; doi:10.1007/s00420-024-02099-3)
Supplement: Supplementary file 1 — Supplementary file1 (DOCX 836 KB) [file 420_2024_2099_MOESM1_ESM.docx]

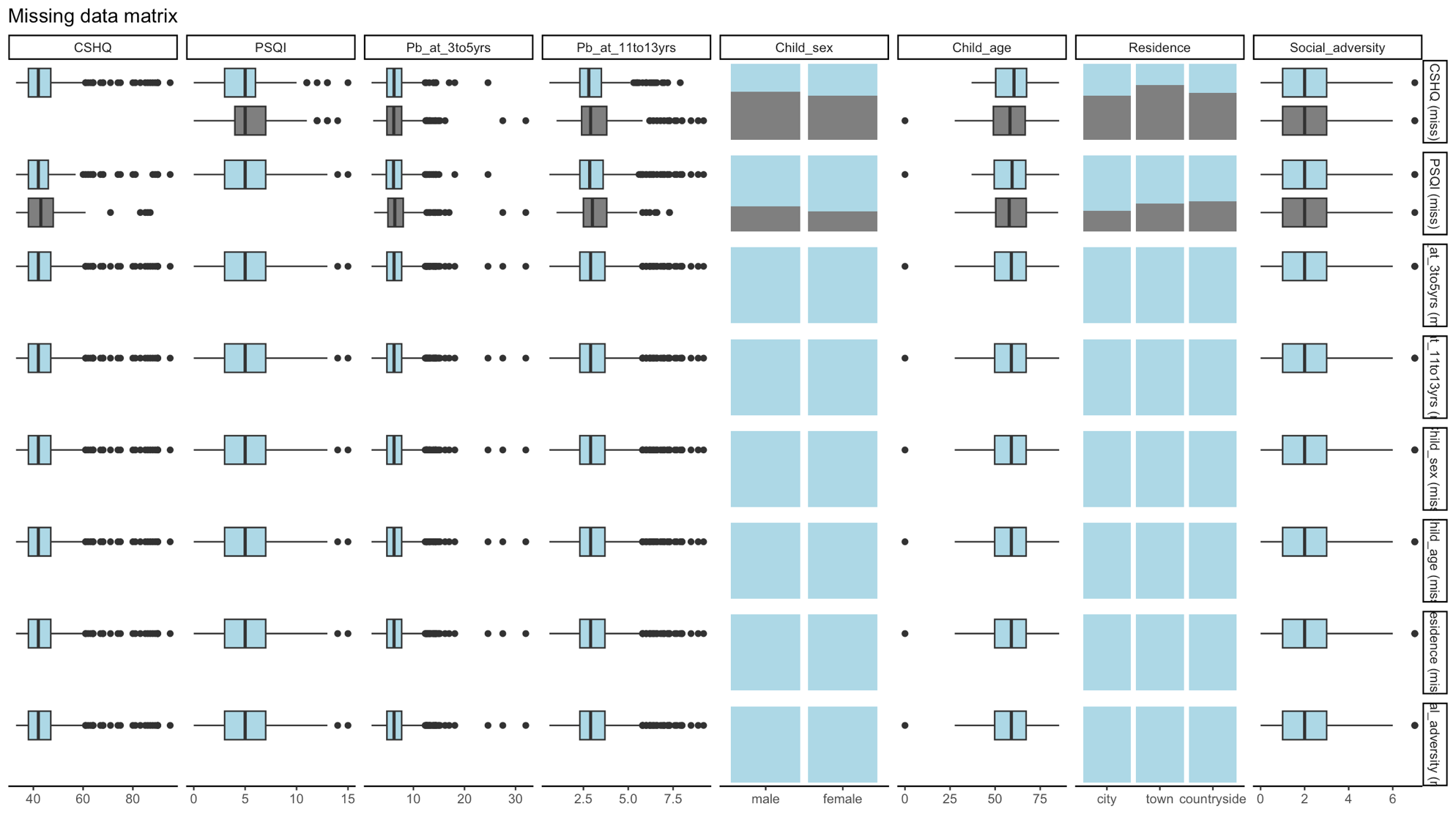


Supplementary Figure 1. Pairwise correlation between missing data and observed data


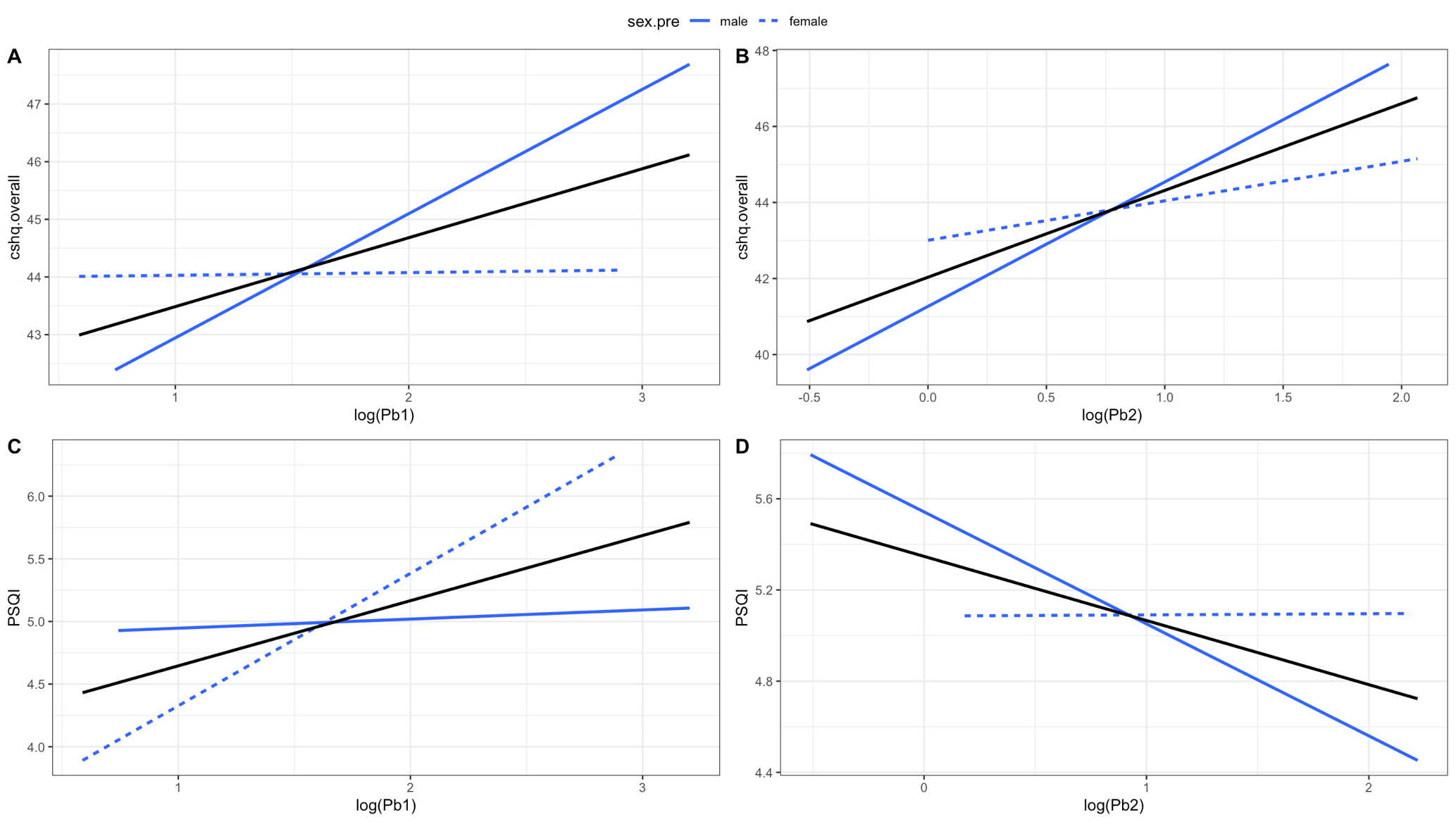


Supplementary Figure 2. BLLs and CSHQ and PSQI overall scores by pre-adolescent sex. Black lines indicate linear regression lines for the overall sample.


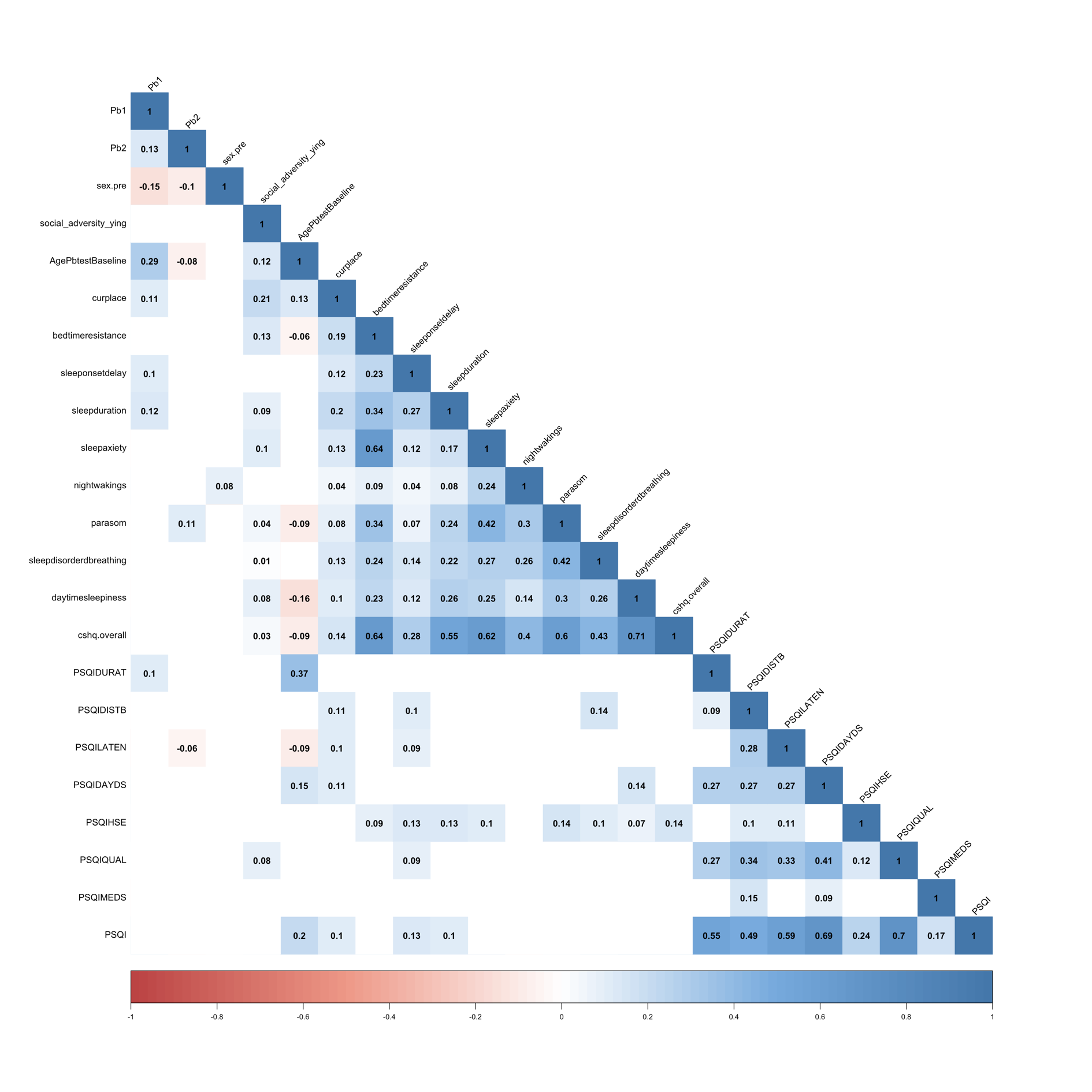


Supplementary Figure 3. Spearman correlation between BLLs and CSHQ (parent-report) and PSQI (adolescent self-report). The blank grid indicates the correlation between variables was not statistically significant (i.e., p $\geq$ 0.05).

Supplementary Tables

Supplementary Table 1 Pattern of Missing data

|  |  | **Not missing** | **Missing** | **p** |
| --- | --- | --- | --- | --- |
| **CSHQ overall score** | | | | |
| Blood lead 3-5 years (mcg/dl) | Mean (SD) | 6.5 (2.7) | 6.5 (2.8) | 0.81 |
| Blood lead 11-13 years (mcg/dl) | Mean (SD) | 3.0 (1.1) | 3.2 (1.2) | 0.048 |
| Sex | Male | 191 (36.7) | 330 (63.3) | 0.081 |
|  | Female | 191 (42.4) | 260 (57.6) |  |
| Age at first blood lead test | Mean (SD) | 58.8 (10.5) | 57.6 (10.9) | 0.091 |
| Residence | City | 298 (42.0) | 412 (58.0) | 0.004 |
|  | Town | 44 (27.8) | 114 (72.2) |  |
|  | Countryside | 40 (38.5) | 64 (61.5) |  |
| Social adversity | Mean (SD) | 2.1 (1.5) | 2.3 (1.5) | 0.044 |
| **PSQI overall score** | | | | |
| Blood lead 3-5 years (mcg/dl) | Mean (SD) | 6.4 (2.5) | 6.8 (3.3) | 0.013 |
| Blood lead 11-13 years (mcg/dl) | Mean (SD) | 3.1 (1.2) | 3.2 (1.1) | 0.384 |
| Sex | Male | 350 (67.2) | 171 (32.8) | 0.05 |
|  | Female | 330 (73.2) | 121 (26.8) |  |
| Age at first blood lead test | Mean (SD) | 58.1 (10.8) | 58.1 (10.6) | 0.974 |
| Residence | City | 517 (72.8) | 193 (27.2) | 0.005 |
|  | Town | 100 (63.3) | 58 (36.7) |  |
|  | Countryside | 63 (60.6) | 41 (39.4) |  |
| Social adversity | Mean (SD) | 2.2 (1.5) | 2.4 (1.4) | 0.107 |

Supplementary Table 2. BLL and preadolescent sleep problems at Wave II (11-13 years) using imputed data (n = 972)

|  | **BLL at 3-5 years** | | | | | | **BLL at 11-13 years** | | | | | |
| --- | --- | --- | --- | --- | --- | --- | --- | --- | --- | --- | --- | --- |
|  | **Total** | | **Female** | | **Male** | | **Total** | | **Female** | | **Male** | |
|  | **β** | **95% CI** | **β** | **95% CI** | **β** | **95% CI** | **β** | **95% CI** | **β** | **95% CI** | **β** | **95% CI** |
| **Parent-report CSHQ** | | | | | | | | | | | | |
| CSHQ Overall | 0.33 | [-0.11, 0.77] | 0.22 | [-0.46, 0.91] | 0.41 | [-0.16, 0.97] | 0.51 * | [0.10, 0.93] | 0.50 | [-0.15, 1.15] | 0.52 | [-0.02, 1.06] |
| Bedtime Resistance | 0.07 | [-0.06, 0.20] | 0.05 | [-0.15, 0.26] | 0.09 | [-0.09, 0.26] | 0.03 | [-0.09, 0.16] | 0.11 | [-0.09, 0.30] | -0.03 | [-0.19, 0.14] |
| Sleep Onset Delay | 0.08 *** | [0.04, 0.11] | 0.06 * | [0.01, 0.12] | 0.09 *** | [0.04, 0.14] | 0.05 ** | [0.02, 0.09] | 0.07 ** | [0.02, 0.13] | 0.03 | [-0.01, 0.08] |
| Short Sleep Duration | 0.14 *** | [0.06, 0.22] | 0.11 | [-0.00, 0.28] | 0.16 ** | [0.06, 0.25] | 0.16 *** | [0.08, 0.23] | 0.17 ** | [0.06, 0.28] | 0.14 * | [0.05, 0.24] |
| Sleep Anxiety | -0.01 | [-0.11, 0.08] | -0.02 | [-0.16, 0.12] | -0.01 | [-0.14, 0.12] | 0.00 | [-0.09, 0.09] | 0.04 | [-0.09, 0.18] | -0.04 | [-0.16, 0.09] |
| Night Waking | 0.01 | [-0.12, 0.15] | -0.03 | [-0.23, 0.17] | 0.05 | ['-0.12, 0.23] | -0.05 | [-0.18, 0.07] | 0.01 | [-0.18, 0.20] | -0.10 | [-0.27, 0.07] |
| Parasomnias | 0.00 | [-0.12, 0.11] | -0.05 | [-0.24, 0.13] | 0.04 | [-0.11, 0.19] | 0.15 ** | [0.04, 0.26] | 0.11 | [-0.07, 0.28] | 0.19 ** | [0.05, 0.33] |
| Sleep Disordered Breathing | 0.02 | [-0.04, 0.08] | 0.01 | [-0.08, 0.10] | 0.02 | [-0.06, 0.10] | 0.05 | [-0.01, 0.11] | 0.02 | [-0.06, 0.11] | 0.07 | [-0.01, 0.15] |
| Daytime Sleepiness (n = | 0.03 | [-0.13, 0.18] | 0.05 | [-0.17, 0.26] | 0.01 | [-0.20, 0.22] | 0.14 | [-0.00, 0.28] | 0.08 | [-0.12, 0.29] | 0.18 | [-0.02, 0.38] |
| **Pre-adolescent self-report PSQI** | | | | | | | | | | | | |
| PSQI overall | 0.02 | [-0.13, 0.17] | 0.14 | [-0.09, 0.36] | -0.08 | [-0.28, 0.11] | -0.02 | [-0.16, 0.12] | 0.01 | [-0.11, 0.32] | -0.11 | [-0.30, 0.07] |
| Short Sleep Duration | 0.00 | [-0.05, 0.05] | 0.00 | [-0.07, 0.07] | 0.00 | [-0.06, 0.06] | 0.02 | [-0.02, 0.07] | 0.08 * | [0.01, 0.15] | -0.02 | [-0.08, 0.04] |
| Sleep Disturbance | -0.01 | [-0.04, 0.02] | 0.03 | [-0.01, 0.07] | -0.05 * | [-0.09, -0.01] | -0.01 | [-0.04, 0.02] | -0.01 | [-0.05, 0.03] | -0.01 | [-0.05, 0.03] |
| Longer Sleep Latency | -0.05 | [-0.10, 0.01] | -0.02 | [-0.10, 0.05] | -0.07 | [-0.13, 0.00] | -0.06 * | [-0.11, -0.01] | -0.02 | [-0.10, 0.05] | -0.09 ** | [-0.16, -0.03] |
| Daytime Dysfunction Due to Sleepiness | 0.02 | [-0.02, 0.07] | 0.05 | [-0.02, 0.12] | 0.00 | [-0.06, 0.07] | 0.01 | [-0.04, 0.05] | 0.01 | [-0.06, 0.08] | 0.01 | [-0.06, 0.07] |
| Lower Sleep Efficiency | 0.02 | [-0.01, 0.05] | 0.04 | [-0.00, 0.08] | 0.00 | [-0.04, 0.05] | 0.00 | [-0.02, 0.03] | 0.00 | [-0.04, 0.04] | 0.01 | [-0.03, 0.05] |
| Lower Sleep Quality | 0.00 | [-0.04, 0.05] | 0.02 | [-0.05, 0.09] | -0.02 | [-0.08, 0.05] | 0.01 | [-0.03, 0.06] | 0.04 | [-0.02, 0.11] | -0.01 | [-0.07, 0.05] |
| Use of Sleep Medication | 0.00 | [-0.03, 0.02] | 0.00 | [-0.03, 0.03] | -0.01 | [-0.04, 0.02] | 0.00 | [-0.02, 0.00] | 0.00 | [-0.03, 0.02] | 0.00 | [-0.03, 0.03] |

Note: All BLLs were log-transformed. All models were based on imputed dataset and controlled for adolescent age at the first blood lead test, adolescent sex, residence, and social adversity index. *** p < 0.001; ** p < 0.01; * p < 0.05.
